# Satisfaction in parturients receiving epidural analgesia after prenatal shared decision-making intervention: a prospective, before-and-after cohort study

**DOI:** 10.1186/s12884-020-03085-6

**Published:** 2020-07-20

**Authors:** Wan-Jung Cheng, Kuo-Chuan Hung, Chung-Han Ho, Chia-Hung Yu, Yi-Chen Chen, Ming-Ping Wu, Chin-Chen Chu, Ying-Jen Chang

**Affiliations:** 1 Department of Anesthesiology, Chi Mei Medical Center, 901 Zhonghua Road, Yongkang District, Tainan City, 710 Taiwan; 2 Department of Medical Research, Chi Mei Medical Center, 901 Zhonghua Road, Yongkang District, Tainan City, 710 Taiwan; 3 Department of Hospital and Health Care Administration, Chia Nan University of Pharmacy and Science, 901 Zhonghua Road, Yongkang District, Tainan City, 710 Taiwan; 4 Division of Urogynecology and Pelvic Floor Reconstruction, Department of Obstetrics and Gynecology, Chi Mei Medical Center, 901 Zhonghua Road, Yongkang District, Tainan City, 710 Taiwan; 5 Center of General Education, Chia Nan University of Pharmacy and Science, 901 Zhonghua Road, Yongkang District, Tainan City, 710 Taiwan; 6 Department of Recreation and Health-Care Management, Chia Nan University of Pharmacy and Science, 901 Zhonghua Road, Yongkang District, Tainan City, 710 Taiwan

**Keywords:** Labor pain, Epidural analgesia, Shared decision-making, Prenatal

## Abstract

**Background:**

The explanation of epidural analgesia by anesthesiologist would often begin after the parturient is admitted to the hospital. Because of labor pain, the decision of receiving epidural analgesia would often be made by the family members, instead of the parturient herself. We aimed to test whether earlier prenatal shared decision-making (SDM) interventions increase parturient’s comprehension and satisfaction of epidural labor analgesia, compared to conventional explanation after labor pain begun.

**Methods:**

During the 28th week of gestation, we provided the SDM parturient health education as well as a leaflet with quick response codes. Scanning the code would link to education videoclips which explained what epidural analgesia is and its advantages and disadvantages. Original routine practice group parturients received explanation of analgesia after admission for delivery. To measure the satisfaction of labor pain service, the accessibility of information, and the communication with medical staff, we designed a questionnaire with reference to (1) Pregnancy and Maternity Care Patients’ Experiences Questionnaire (PreMaPEQ), (2) Preterm Birth Experience and Satisfaction Scale (P-BESS), and (3) Women’s Views of Birth Labor Satisfaction Questionnaire (WOMBLSQ). The questionnaire was amended after a pretest involving 30 parturients who had received epidural analgesia. Scree test analysis and exploratory factor analysis were performed; then, the questionnaire was revised again. A total of 200 valid questionnaires were collected—100 each from the original routine practice group and the SDM group.

**Results:**

The SDM group reported significantly higher satisfaction with and understanding of epidural analgesia, and a significantly higher satisfaction with the information received, and the quality of pain relief. After SDM intervention, significant increasement of the average satisfaction scores in question “my epidural is effective” (9.10%; mean difference: 0.38; 95% confidence interval, 0.17 ~ 0.59; *p* < 0.001) and “The effect of epidural is just as what I have expected” (10.41%; mean difference: 0.41; 95% confidence interval, 0.18 ~ 0.64; *p* < 0.001) was demonstrated.

**Conclusions:**

An earlier prenatal SDM intervention with sufficient information through videoclips increased parturients’ comprehensions and satisfaction of epidural analgesia service.

**Trial registration:**

ISRCTN registry, 14,256,563. Registered April 1st, 2020 (10.1186/ISRCTN14256563).

## Background

Labor pain is very stressful for many birthing mothers. Conventionally, the anesthesiologist explains the benefits and risks of epidural labor analgesia on maternal request when the parturient has admitted to the hospital for delivery and mostly labor pain has begun. Because distraction by labor pain, the decision of receiving epidural analgesia would often be made by the family member, such as her husband, instead of the parturient herself. On the other hand, although most of the parturients have good decision-making ability before the distraction of labor pain, she might not receive insufficient professional information provided by anesthesiologists. We hypothesized that the parturient would capture sufficient information about the options of epidural analgesia and would have higher satisfaction of pain service, if adequate education and discussion provided prenatally before her labor begins.

In order to improve the quality of medical care, this study aimed to compare the satisfaction of and the level of comprehension in epidural analgesia between parturients that received the original routine practice (original group) and those that received prenatal shared decision-making (SDM) [1–3] principal-based health education intervention, assisted by educational film provided by the anesthesiologist, started on their regular return visit during their 28th week of gestation (SDM group).

## Methods

### Inclusion and exclusion criteria

This present “before and after” designed questionnaire study was conducted during June 14, 2018 to December 25, 2018, after the approval of the Chi Mei Medical Center Institutional Review Board (IRB: 10705–010).

We included parturient aged ≧20 years and had used epidural analgesia during the natural birth process, and can read Chinese or communicate in Mandarin or Taiwanese.

Women comorbid with mental, emotional, or psychological disorders, regardless of whether they are receiving psychiatric medication, were excluded from our study. We also excluded those women with a history of drug addiction or drug dependence of analgesics, for example, had a history of using morphine daily for more than 30 mg of oral morphine equivalent for more than 6 weeks, from our study. Women who are in the intensive care unit after delivery were also excluded.

### Questionnaire

Because we did not find any ideal and validated questionnaires in Chinese to calculate the differences in satisfaction and comprehension [4], we therefore designed a questionnaire referenced from existed valid English questionnaires. First, we adapted questions from (1) the Pregnancy and Maternity Care Patients’ Experiences Questionnaire (PreMaPEQ) [4], (2) Satisfaction Scale (P-BESS) [5], (3) Women’s Views of Birth Labor Satisfaction Questionnaire (WOMBLSQ) [6, 7]. These referenced questionnaires are publicly available and have assured validity and reliability.

The second step was modifying the adapted questions to fit specifically to epidural analgesia scenario (Additional file 1) and translating them into Chinese that are understandable for elementary educated level parturients. The team of obstetrics and gynecology (OBGYN) anesthesia specialists, director of obstetrician department, and a professional English teacher in Chi Mei medical center accomplished this step together, and confirmed that the Chinese version had reached the original semantics. Following translation of the questionnaire, it was tested on volunteers with an elementary level of reading. The above questionnaire, which has six different categories, was the version 1 questionnaire in our research (Additional file 2). The six categories were: 1. Healthcare communication, 2. Labor pain, 3. Overall satisfaction, 4. Access to information, 5. Decision, 6. Expectation and reality.

### Pilot study

In the third step, we tested the version 1 questionnaire to 30 parturients that had received epidural analgesia as pre-test pilot study, in order to confirm whether version 1 questionnaire had sufficient reliability and whether it had reserved the essence of the questionnaires we quoted from, and to detect flaws and weakness of version 1 questionnaire. All of the questions were answered based on the following five-point ordered response scale: 1 = Strongly disagree, 2 = Disagree, 3 = Neither agree nor disagree, 4 = Agree and 5 = Strongly agree [4, 8].

In the fourth step, we calculated the reliability of the version 1 questionnaire using these 30 returned version 1 questionnaires. Standardized Cronbach’s alpha of version 1 questionnaire was 0.86 and the Variance of explanation was 76.99%, indicating that the version 1 questionnaire had good reliability.

Because we hoped to compose a satisfaction assessment that has enough efficiency and reliability with the least number of categories and questions, in the fifth step, we entered the results of the 30 “Pre-test” version 1 questionnaires into the standard statistics software SPSS (Statistical Product and Service Solutions) software (version 19.0, Chicago, IL) to perform Scree test analysis. The turning point of the Scree test corresponded to five categories, that is, the questionnaire could be simplified into five categories, and the characteristics of the questionnaire could still be maintained. Each category contained at least 3 questions and should load significantly to ensure all of the subscales to be successfully identified [9]. Therefore, we dispersed all the questions according to exploratory factor analysis (EFA) (Additional file 3). Questionnaire questions were entered into principal axis factoring analyses. EFA results were interpreted as supportive if loadings exceeded 0.60. After vertical rotation and factor loading calculation, the questions were constructed to five categories based on theoretical structure processing. The questions grouped according to the structure and process categories. We obtained the version 2 questionnaire by the EFA result. This version 2 questionnaire had 5 categories based on the above-mentioned statistical methods (Additional file 4).

The team of OBGYN anesthesia specialists, including Professor Chu, doctor Ying-Jen Chang, Chia-Hung Yu, and Wan-Jung Cheng, who is responsible for introducing epidural analgesia revised the version 2 questionnaire according to the clinical viewpoint:

1. Retained questions of similar clinical significance in the same category.
2. Performed data reduction by deleting questions with low factor loadings.

After the revision and the re-naming of each category, the version 3 questionnaire was developed.

The sixth step was to calculate the reliability of the version 3 questionnaire using pre-test results (Additional file 5). The statistician again implemented the new vertical rotation and EFA. The analysis results of the version 3 questionnaire were as follows:

1. Reliability: Standardized Cronbach’s alpha of the version 3 questionnaire was 0.891.
2. The Variance of explanation was 84.7%.
3. Meyer-Olkin (KMO) value is 0.852 and *P*-value of Barlett’s index <.0001.

Based on the above, the version 3 questionnaire was credible, effective, and the unexplained error was small enough for large-scale distribution. The version 3 questionnaire can be applied to further test large scale epidural analgesia education and communication survey. The seventh step was to rename the outline of each category. *Since our prenatal education protocol was set under the concept of SDM, categories of our final questionnaire was named base on the SDM model* [10–14]*. The three critical steps were: 1. Team talk* [11], providing high quality information, making sure that parturients know that reasonable options are available. 2. *Option talk*, refers to providing more detailed information about options and support parturients to deliberate and integrating about their options. 3. *Decision talk*, refers to supporting the work of exploring preferences and deciding what is best. The team of OBGYN anesthesia specialists named each category as:

1. Team Talk
2. Option Talk
3. Decision Talk
4. The Expectation of Epidural Analgesia
5. Overall Satisfaction

**Fig. 1:**
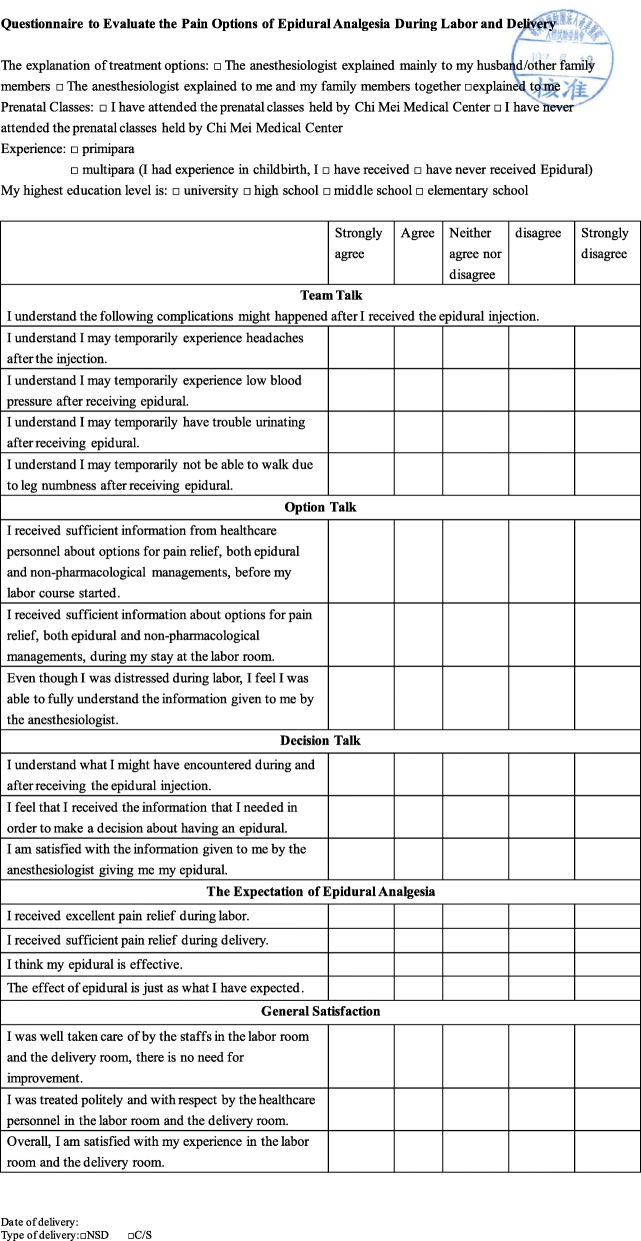
The final questionnaire

Since the final questionnaire was designed to test the SDM intervention, the outline reflected the different phases of the healthcare course in the SDM concept. After renaming, the version 3 questionnaire can then be used as the final version. The final questionnaire is shown in Fig. 1.

### Partriuent grouping

**Fig. 2:**
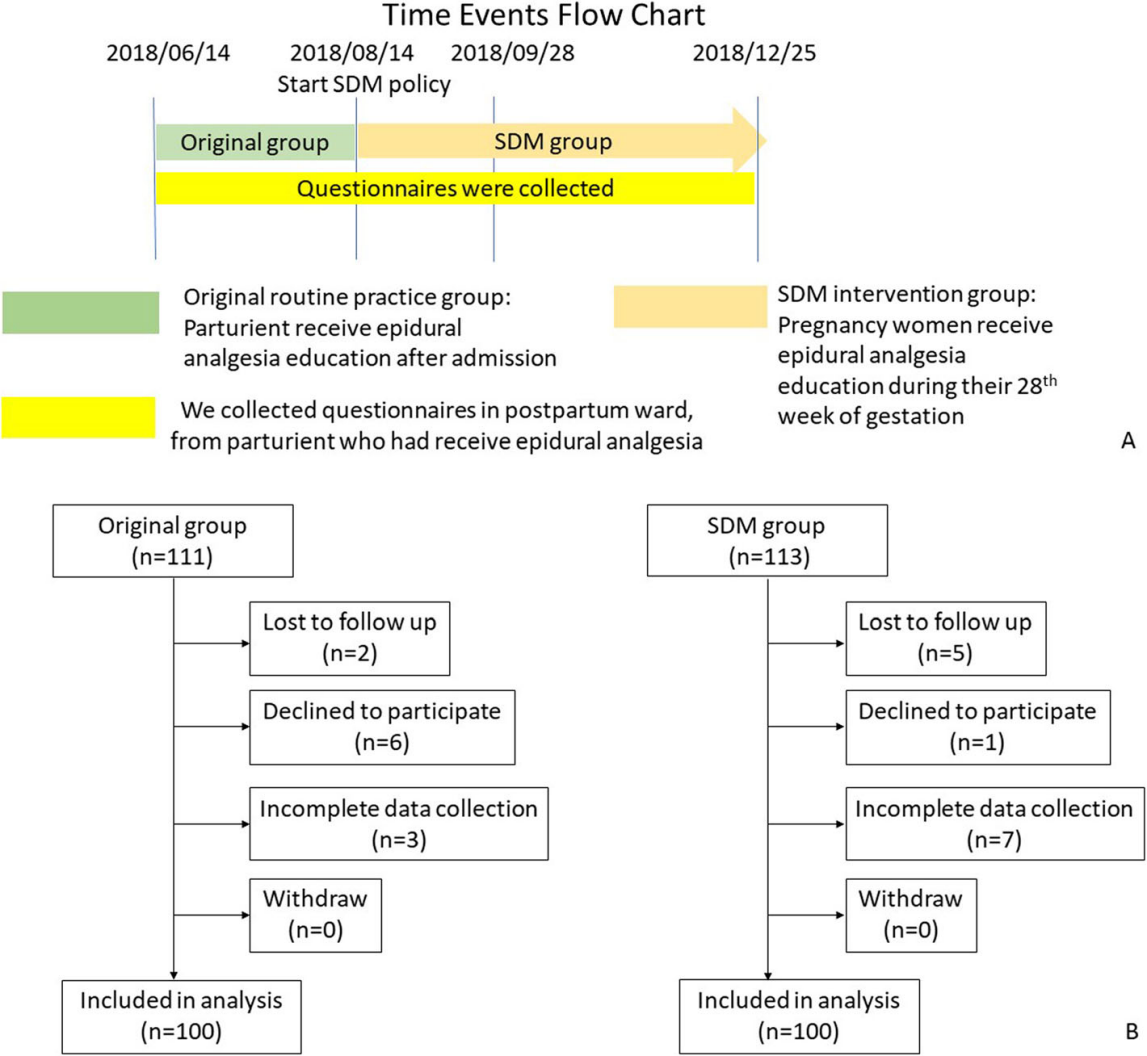
The participant flow chart **a** The time events flow chart **b** The participant enrollment. SDM: shared decision-making

This is a “before and after” study. This study took place between June 14, 2018 and December 25, 2018, allowing a comparison period before and after the change in prenatal SDM protocol (Fig. 2). Before the implementation of the prenatal SDM policy on 14 August 2018, we evaluated the parturient and explained the procedure, risk/benefit and possible complication to the parturient and family member at the delivery room, on the request of labor pain service. All parturients were assigned as original routine practice group (original group) before 14 August 2018 (Fig. 2a). After the prenatal SDM policy implementation on 14 August 2018, we offered above explanations and education at 28 gestation weeks, when she had a prenatal visit. All parturients were assigned as SDM group after the SDM policy been implemented.

### Description of the intervention of SDM

**Fig. 3:**
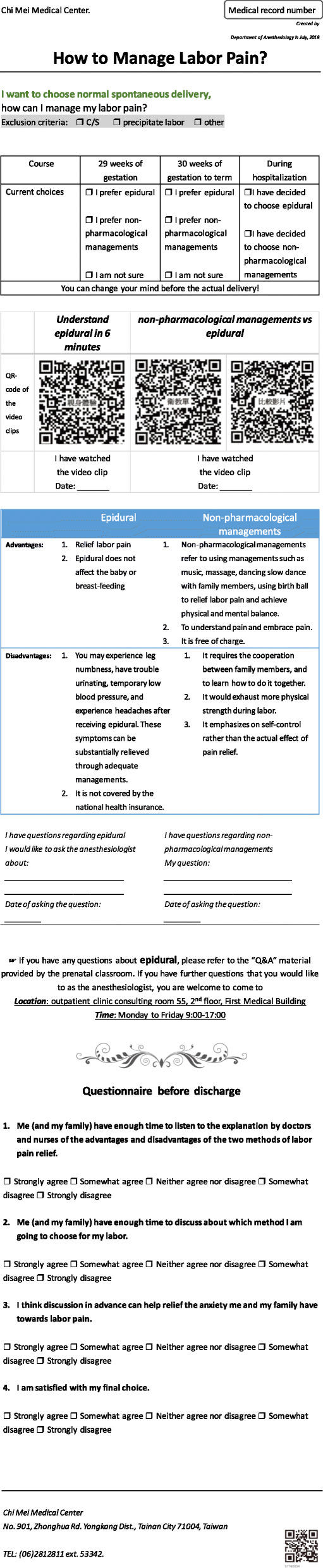
Health education leaflet with the QR code of the health education videos, comparing epidural analgesia and non-pharmacological labor pain managements. Parturient can watch the health education videos on the smartphone via QR code, and compare the advantages and disadvantages of epidural analgesia and non-pharmacological managements such as music and massage. QR code: Quick Response code. SDM: shared decision-making

At parturient’s return visit during their 28th week of gestation, the registered nurse in the prenatal classroom provided the health education as well as a leaflet with Quick Response (QR) code of the health education videos on it, so that parturient can watch the videos on the smartphone, and compare the advantages and disadvantages of epidural analgesia and non-pharmacological managements such as music and massage. The SDM group received this education leaflet on their return visit during their 28th week of gestation. All of the education program administered to the parturients is in Chinese and had been modified so that parturients with elementary school level of education can understand. English translation of the health education leaflet, which is shown in Fig. 3, is only translated for the publication of this research.

The advantages of SDM are as follows: 1. Parturients have the leaflet with the QR code of health education videos, and the videos can be played, paused, and replayed at any time according to needs. 2. Because parturients can watch the films and learn about epidural analgesia in advance, they have time to consult the anesthesiologist about epidural analgesia before her labor begin. 3. Family members of the parturients can watch the films as well, so parturients can discuss medical decisions with their families. Every concerned family member can participate in the decision-making process and understand the possible complications of this medical intervention and how effective the epidural analgesia will be. This can also ensure neither the physician nor any single family member is making this decision for the parturient. 4. Parturient and family members have time to digest the information. With enough information, they would not have unrealistic expectations for epidural analgesia and this can help reduce medical disputes. We collected the questionnaire in postpartum ward, a comparison period before and after the apply in prenatal SDM protocol. The questionnaire evaluating the understanding about epidural analgesia before the process, and the satisfaction on the actual process of receiving epidural analgesia. Then we compared the scores between these two groups.

### Study performance

The corresponding author, issue the questionnaire and recruited the parturient after labor pain service since June 14, 2018 to December 25, 2018. The recruitment took place in postpartum ward.

### Sample size estimation

The sample size for the study was based on the experiences of the PreMaPEQ study [4]. Based on the assumption that the satisfaction score would be increased by 10% after SDM intervention, the sample size was predetermined using a power analysis with a significant level of α = 0.05 (one-sided) and power of 1 − β = 0.8. The results indicated that 85 parturients would be needed for each group. Allowing for potential drop-outs, we evaluated 111 parturients in original group and 113 in SDM group, after exclusion, it left 100 parturients in each group. Taking into account of incomplete or nonassessable files, the target number of subjects was 200 enrolled parturients (100 parturients/group). (Table 1).

**Table 1:** The comparison of questions in PreMaPEQ and our questionnaire for power calculation

Question	The mean value of PreMaPEQ version	The standard deviation value of PreMaPEQ version	Number of Questionnaires required to have power greater than 0.8	The power of the presenting research data (100 questionnaires)
PreMaPEQ version: Did you receive sufficient information about options for pain relief during the birth?	3.5	1.2	48	> 0.999
PreMaPEQ version: Did you receive information about who had the main responsibility for you?	3.6	1.3	85	0.857

For power evaluation, the representative questions in our study were compared with the similar ones in the already valid Pregnancy and Maternity Care Patients’ Experiences Questionnaire (PreMaPEQ) [4]

### Statistics

All of the questions were answered based on the following five-point ordered response scale: 1 = Strongly disagree, 2 = Disagree, 3 = Neither agree nor disagree, 4 = Agree and 5 = Strongly agree. We assess the improvements in scores by “Percentage of score increasement”, which was calculated as:

$$ \left(\mathrm{score}\ \mathrm{of}\ \mathrm{SDM}\ \mathrm{group}-\mathrm{score}\ \mathrm{of}\ \mathrm{original}\ \mathrm{group}\right)/\mathrm{score}\ \mathrm{of}\ \mathrm{original}\ \mathrm{group}. $$

We used the exploratory factor analysis (EFA) to construct the validity, and KMO or Barlett’s index to determine the reliability of the questionnaire. The questionnaire contained 17 questions, and was categorized into 5-group questions related to team talk, option talk, decision talk, satisfaction of labor pain treatment and overall service. The categorical variable was tested by Pearson’s chi-square and continuous variables by T-test. We set the significance level at 0.05. All the score difference between SDM group and original group was calculated from Welch’s T-test. The 95% confidence interval was performed using unequal variance in two groups. We used SPSS (version 19.0, Chicago II) to perform the statistical analysis.

## Results

A total of 224 questionnaires were collected in this study, of which 200 were valid questionnaires (Fig. 2b). Of the 200 questionnaires, 100 were from subjects receiving original routine practice, and 100 were from subjects receiving SDM intervention. The raw data of the 200 questionnaires were displayed in Additional file 6.

Table 2 demonstrated the baseline characteristics were not different between 2 groups in age, parity and education levels etc. Most of the parturients in both groups are primiparas. Respectively, 68.4 and 80% of the multiparous women in original and SDM group, had previous experience of labor epidural analgesia (*p* = 0.391). Our results also showed that over 80% of the parturients in both groups had university degree or above. The only different was the cesarean section(C/S) rate for those had epidural labor analgesia, which was significant lower (*P* = 0.008) in the SDM group (28%) than that of original group (40%).

**Table 2:**
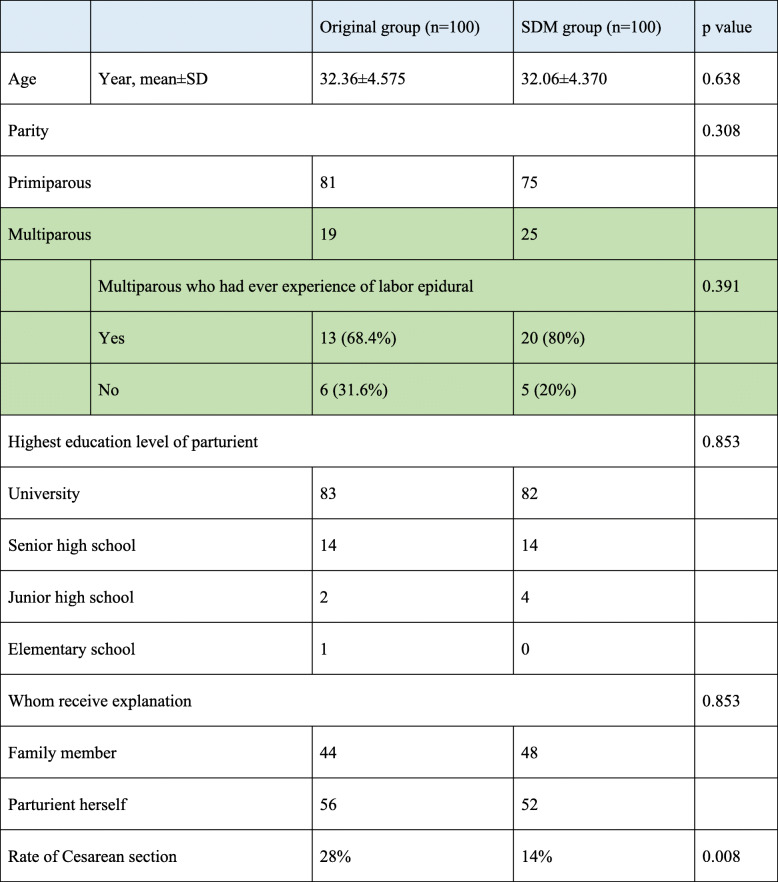
Subjects’ baseline characteristics stratified by interventions

Table 3 showed the mean score on each question for the two groups, and the percentage of score increasement on each question for parturients had SDM intervention. SDM group had higher score in all questions regarding satisfaction and comprehension of possible complications of epidural analgesia. About the comprehension of possible complications, the understanding of “temporarily headaches might happen” showed significantly higher in SDM group than that of original group [mean difference (MD): 0.22; 95% confidence interval (CI): 0.01 to 0.43; *p* = 0.022]. Although, SDM parturients scored higher to questions about the understanding of the possibilities of “temporarily low blood pressure”, “having trouble urinating”, and “not able to walk due to leg numbness”; however, not reaching a statistical significance in MD.

**Table 3:**
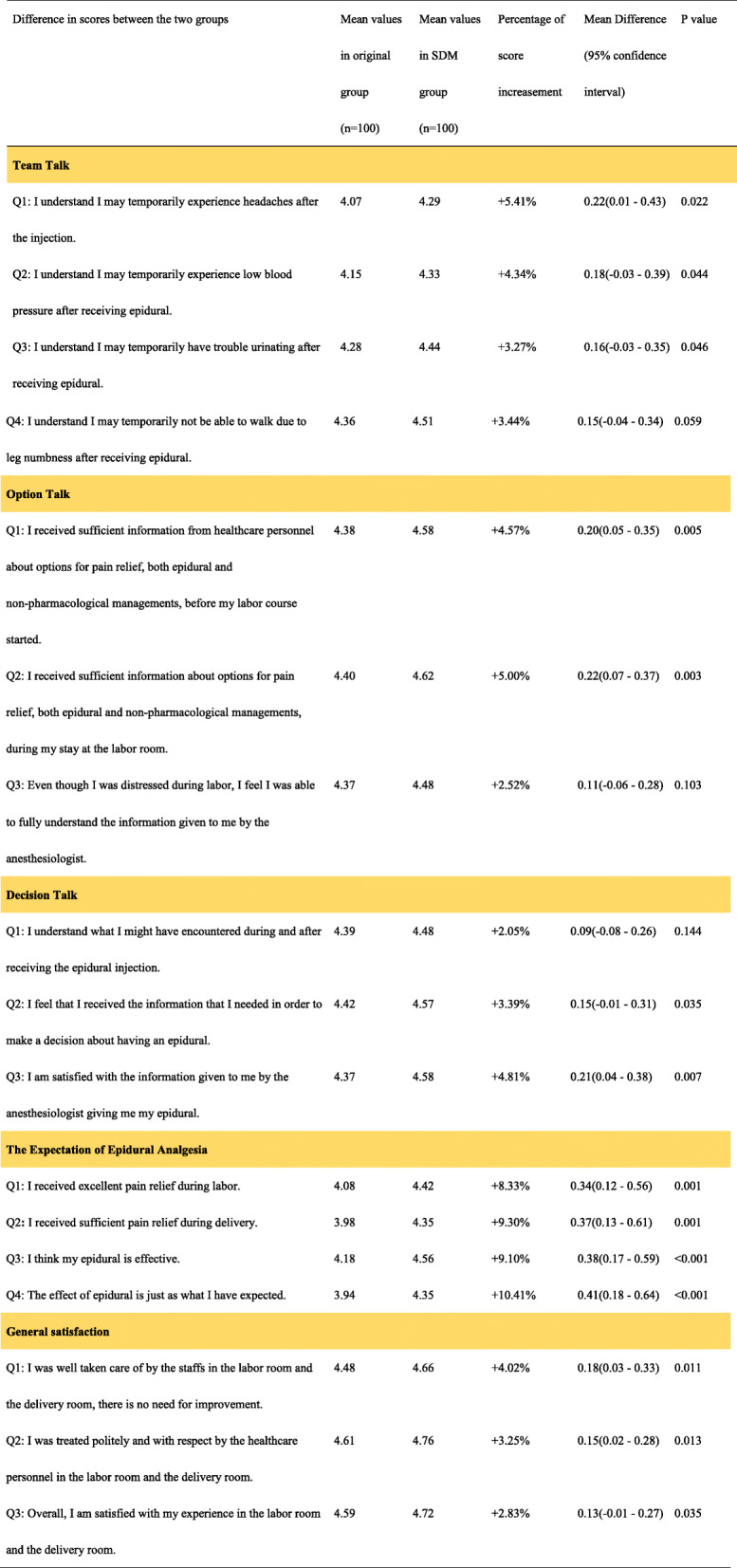
Percentage of score increasement in the comprehension^a^ and satisfaction^b^ between study groups

The scoring of questions related to satisfaction of information providing, such as” I received sufficient information about options for pain relief”,” before labor course started” and “stay at the labor room”, were all higher in SDM groups. (Table 3).

As to the question related to decision talk,” the information given by whom give me epidural”, it showed significant higher scores in SDM groups (MD 0.21; 95% CI, 0.04 to 0.38; *p* = 0.007).

In addition, SMD group parturients all gave significant higher scores to these questions related to the expectation of epidural analgesia, including “excellent pain relief during labor” (MD: 0.34; 95% CI: 0.12 to 0.56; *p* = 0.001) and” sufficient pain relief during delivery” (MD:0.37; 95% CI: 0.13 to 0.16; *p* = 0.001). The scoring of questions about the expectation and reality of labor analgesia, for example, the question” my epidural analgesia is effective” (MD: 0.38; 95% CI,0.17 ~ 0.59; *p* < 0.001), and question” epidural is just as what I have expected” (MD: 0.41; 95% CI,0.18 ~ 0.64; *p* < 0.001), showed a lower expectation gap in SDM group than that of the original group.

As the question” I was well taken care of by the staffs, there is no need for improvement” (MD: 0.18; 95% CI,0.03 to 0.33; *p* = 0.011), and “I was treated politely and with respect “(MD: 0.15; 95% CI,0.02 to 0.28; *p* = 0.013), indicating higher satisfaction score for SDM group about the service in the labor room and the delivery room.

The most significant increasement in scores after the SDM intervention were answering to questions “I think my epidural analgesia is effective” (increasement 9.10%) and “The effect of epidural is just as what I have expected” (increasement 10.41%).

## Discussion

To the best of our knowledge, this is the first prenatal SDM concept-applying research in parturients for improving the comprehension and satisfaction of epidural analgesia. We found the benefit of setting an earlier epidural analgesia education protocol before labor pain is in progress. Thus, parturient may have enough time to discuss with her family and anesthesiologist about the individual concern before making the final decision of receiving epidural analgesia or not. These earlier education and communication will eventually increase the satisfaction of labor analgesic service and promote the harmony between parturients and health care-givers.

### SDM model is appropriate to pregnancy woman

Several studies reported that parturient having epidural analgesia does not often receives enough information before the procedure was conducted [15–18]. Since parturients often do not have the opportunity to personally discuss with the doctor, they may feel “whether to receive epidural analgesia or not” is not for her to decide [17] and losing their own autonomy [17]. In order to improve maternal welfare, we promoted the use of epidural analgesia leaflet, which included a QR code of a health education video made by anesthesiologists. Based on the following, the SDM program is particularly suitable for pregnancy woman. First, most parturients have university degrees in our study(Table 2), which might be a very good target population for a successful SDM intervention, because SDM by definition requires people to have enough comprehensive ability to understand the medical process in order to be able to discuss with the doctor their desired method of interventions [19, 20]. Second, childbearing women were in relatively young age among both groups. Young people often have the ability to use smartphone to watch videos via QR code [21]. Third, pregnant women have to schedule prenatal visits every 2 weeks after their 28th week of gestation; therefore, they have oppurtunities to discuss with anesthesiologist in their routine visits in hospital. Fourth, if the education started before labor begins, partriuent who had special physical condition, such as morbid obesities [22], could have more time to discuss with her anesthesiologist. Due to the above reasons, SDM model is particularly appropriate to implement on pregnant woman.

### Advantage of video-assisted education

The benefits of using a video-assisted education is as follows: 1. The film can describe the procedure steps in details [19]. 2. Using film is more realistic than using pictures [23]. 3. The film relieved the medical staffs from time-consuming repeated explanations [24]. 4. The film can be paused and played at any time according to the need of the viewer, and the parts that were not understood can be repeated. It can also be circulated among family members, so that every concerned family member can participate in the decision-making process [25].

In today’s medical settings, most parturients have a feeling of lacking some information if the doctors only communicate by words [19, 26]. Literature has confirmed that the information gained by film plus face-to-face visits is greater than that gained by health education leaflet plus face-to-face interviews and face-to-face interviews alone [19]. An U.S. research in Oregon has demonstrated that an education program consisted with a video increased epidural analgesia use in non-English speaking parturient [27]. However, the above study did not set a protocol of education.

According to previous research, the use of video has been proven to reduce anxiety before general anesthesia [23]. They used the VAS score: 1–10 points to evaluate the anxiety index before and after watching the video of detailed general anesthesia risk education in the clinic, and the anxiety index of the parturient dropped significantly after watching the film [23]. The same conclusion can be obtained using the validated anxiety index questionnaire, the State-Trait Anxiety Inventory (STAI). Presumably, the reason for this may be that a detailed and visualized introduction of the general anesthesia process can reduce the fear of the unknown in parturients receiving general anesthesia for the first time [19]. The STAI study also proved that there exists a positive correlation between lowering anxiety and good outcome [23]. The process of anesthetic consultation of epidural analgesia is similar to the consultation of general anesthesia. **“**The effect of epidural is just as what I have expected” showed significant higher score in SDM group than original, might provide some evidence for prenatal SDM reducing the fear of the unknown. Base on the above, health education film is a better way of accessing information than traditional face-to-face interviews alone.

### Understanding of potential complications

As far as intervention is concerned, everyone should know the course and risk of any procedure [23]. An Irish study revealed that most of the postpartum parturient, who had received epidural analgesia, don’t know what complication might occurred after the epidural analgesia for labor. The Irish study demonstrated that less than 30% of them are aware of the most common complications [28]. The comprehension of temporarily headaches, low blood pressure, and have trouble urinating may happen after receiving epidural is improved in our SDM group than original (Table 3).

### SDM enhances the understanding of complications

Knowing the procedure well including understanding possible complications, as part of a medical treatment [29, 30], may sometimes happen. According to previous study, all parturients can accept the complication of post dual puncture headache if they are explained before it happen [30–34]. In fact, although the rate of complications is independent from the level of explanation before interventions, some evidence showed increased preoperative satisfaction will decrease postoperative complications [19]. It is reasonable to speculate that the incidence and severity of complications is similar, but a detailed explanation before intervention can reduce mistaking some adverse effect, such as leg numbness, as complications. Previous questionnaire study confirmed that parturients want the doctor to explain all the common conditions after epidural analgesia, including headache, hypotension, difficult voiding, inability to walk due to numbness, and poor analgesic effect [17]. A detail explanation of epidural injection can let parturient get a clearer picture of what might happen after the procedure.

### SDM eliminates a disparity between expectations and reality of labor analgesia

Labor pain is subjective so it is different from person to person. However, it is important to let pregnant women know how effective the epidural analgesia will be. After prenatal education, parturient will understand that a heavy dose of epidural analgesia could ease the pain but would also weaken the muscle of the lower part of body, such as the pelvic muscle, which might delay the labor process [32]. Although parturients wish they could walk after the epidural analgesia, but in fact some of them can’t walk due to numbness [35]. For preserving the lower body muscle power, anesthesiologists will adjust the dose of epidural analgesia to ease the pain but not to complete resolve the pain. In a previous study, 21% of the parturients did not know enough before receiving the epidural analgesia, after giving birth, they thought that their epidural analgesia didn’t work [18]. 26% of the parturients said they did not know what the benefits of epidural analgesia were [18]. After understanding the pain can only be lessened but not total pain-free, in order to preserve pelvic muscle power, the reality and expectations will be closer. Parturients were generally satisfied with the effect of epidural analgesia in the SDM group, and that they considered their epidural analgesia to be more effective than original group. For example, the scores significant increase in question “I think my epidural is effective” and “The effect of epidural is just as what I have expected” after SDM interventions.

We also notice a higher pain tolerance in SDM group than original. Our question such as” I received sufficient pain relief during delivery” and” I received excellent pain relief during labor” showed some evidence that the smaller the gap between expectation and reality is, the higher pain tolerance she would have [36].

Moreover, the rate of C/S after receiving labor epidural analgesia was significant lower in the SDM group (original: SDM: 28%: 14%, *p* = 0.008) (Table 2). A possible reason of lower C/S rate is that the patruients in SDM group had been well educated and therefore had higher tolerance for pain compared to that of the original group. However, we need to do more research to confirm the above hypothesis.

### SDM increases satisfaction

Previous study showed once parturients are admitted, “maternal participation in anesthesia decision” is an important key to satisfaction [23]. According to previous studies, if everything is well explained before surgery, the overall postoperative satisfaction might be higher [26, 37, 38]. In our SDM group, general satisfaction is high.

SDM group showed satisfaction score not only improved towered the anesthesiologist who perform epidural injection but also the overall impression on the delivery room. For example, SDM group had higher score in the question of “I am satisfied with my experience in the labor room and the delivery room”, “I was well taken care of by the staffs in the labor room and the delivery room, there is no need for improvement”, and “I was treated politely and with respect by the healthcare personnel in the labor room and the delivery room”.

### Limitation

This study has several limitations. First, some of the laboring mothers, who have had prenatal SDM education, but eventually did not have epidural labor analgesia, were excluded from the study. Although, they might answer those questions about SDM talks very well. This questionnaire contains questions about satisfaction of the labor analgesia, which these parturients cannot answer. Second, the C/S rate was significant higher in original group than that in SDM group. One of the possible explanations is that SDM parturient did not had an overly-high expectation of the analgesic effect, therefore, did not request a C/S so frequent as original group, instead of C/S by clinical indication. However, this speculation cannot be confirmed, because we did not record of the reason of C/S. Third, since this is a single hospital study, and popularization of higher education in Taiwan, our results might not be generalized to other developing or under-developing countries.

## Conclusion

Prenatal sharing decision-making interventions supported by online educational video may contribute to maternal understanding and satisfaction with epidural labor analgesia services. We recommend that further studies be conducted in more countries, especially those that higher level of education not so popularized, for confirming whether earlier SDM intervention before labor promotes maternal understanding and satisfaction with epidural analgesia.

## Supplementary information

**Additional file 1.** The references of our questions. Questions in our study and where it adapted from.

**Additional file 2.** The Version 1 questionnaire. This is the pre-testing questionnaire administered to the parturients, and the English version is only translated for the publication of this research.

**Additional file 3.** Exploratory factor analysis results. Exploratory factor analysis results of all the version 1 questionnaire questions after pre-testing.

**Additional file 4.** The Version 2 questionnaire. This is the English version of Version 2 questionnaire, whose categories were based on the above-methodology chapter-mentioned statistical method.

**Additional file 5.** Exploratory factor analysis results. Exploratory factor analysis results of all the version 3 questionnaire questions after pre-testing.

**Additional file 6.** The raw data. The raw data of the 200 file-questionnaires.

## Data Availability

All data generated or analyzed during this study are included in supplementary information: Additional file 6. The detail datasets used and analyzed during the current study are available from the corresponding author on reasonable request.

## Abbreviations

SDM: Shared decision-making
SDM group: SDM intervention group
original group: Original routine practice group
OBGYN: Obstetrics and gynecology
EFA: Exploratory factor analysis
QR code: Quick Response code
PreMaPEQ: Pregnancy and Maternity Care Patients’ Experiences Questionnaire
P-BESS: Preterm Birth Experience and Satisfaction Scale
WOMBLSQ: Women’s Views of Birth Labor Satisfaction Questionnaire
MD: Mean difference
C/S: Cesarean section
CI: Confidence interval
