# Supplementary material for: Satisfaction in parturients receiving epidural analgesia after prenatal shared decision-making intervention: a prospective, before-and-after cohort study
Source: BMC Pregnancy Childbirth. 2020 Jul 20;20:413. doi: 10.1186/s12884-020-03085-6 (PMC7370438; doi:10.1186/s12884-020-03085-6)
Supplement: Supplementary file 1 — Additional file 1. The references of our questions. Questions in our study and where it adapted from. [file 12884_2020_3085_MOESM1_ESM.doc]

| **Questions in our study, after fitting specifically to** **epidural analgesia scenario** | **The adapted question, from referenced questionnaire** | **Referenced questionnaire** |
| --- | --- | --- |
| I understand I may temporarily experience headaches after the injection. | How important it is to you that you be informed about the existence of the risk of headache? | ERCQ*. Question 17 |
| I understand I may temporarily experience low blood pressure after receiving epidural. | How important it is to you that you be informed about the existence of the risk of lowered blood pressure? | ERCQ. Question 20 |
| I understand I may temporarily have trouble urinating after receiving epidural. | How important it is to you that you be informed about the existence of the risk of inability to pass water? | ERCQ. Question 21 |
| I understand I may temporarily not be able to walk due to leg numbness after receiving epidural. | How important it is to you that you be informed about the existence of the risk of inability to walk during labour? | ERCQ. Question 28 |
|  | How important it is to you that you be informed about the existence of the risk of paralysis? | ERCQ. Question 25 |
| I received sufficient information from healthcare personnel about options for pain relief, both epidural and non-pharmacological managements, before my labor course started. | Did you receive sufficent information about options for pain relief during birth. | PreMaPEQ. Question 30 |
|  | I believe that women should receive information about the option of epidural anesthesia well before labour begins. | ERCQ. Question 54 |
| I received sufficient information about options for pain relief, both epidural and non-pharmacological managements, during my stay at the labor room. | Did you receive sufficent information about options for pain relief during birth. | PreMaPEQ. Question 30 |
|  | Did you revive suffient information during your stay at the delivery ward? | PreMaPEQ Question 57 |
| Even though I was distressed during labor, I feel I was able to fully understand the information given to me by the anesthesiologist. | Even though I was distressed during my labour I feel I was able to fully understand the information I was given regarding the epidural. | ERCQ. Question 56 |
| I understand what I might have encountered during and after receiving the epidural injection. | The staff explained to me what would happen during the birth. | P-BESS. Question 2-4 |
|  | The staff explained to me what would happen to my baby when he/she was born. | P-BESS. Question 2-3 |
| I feel that I received the information that I needed in order to make a decision about having an epidural. | In general, I feel that I received the information that I needed in order to make a decision about having an epidural. | ERCQ. Question 58 |
|  | Did you wish to be invloved in the decisions concering your birth? | PreMaPEQ. Question 62 |
|  | Were you involved in decisions concering your birth? | PreMaPEQ. Question 63 |
|  | I didn't want to have an epidural but felt I was forced into having one by other people. | ERCQ. Question 59 |
| I am satisfied with the information given to me by the anesthesiologist giving me my epidural. | Did you receive information from who had the main responsibility for you? | PreMaPEQ. Question 61 |
|  | How satisfied are you with the information you were given by the doctor giving your epidural? | ERCQ. Question 57 |
| I received excellent pain relief during labor. | I got excellent pain relief in labour. | WOMBLSQ. Question 20 |
|  | How pleasant did you find the experience of having the epidural inserted? | ERCQ. Question 10 |
| I received sufficient pain relief during delivery. | Did you receive sufficient pain relief during labor? | PreMaPEQ. Question 64 |
| I think my epidural is effective. | How severe was your labour pain prior to receiving your epidural? | ERCQ Question 5 |
|  | How severe was your labour pain after receiving your epidural? | ERCQ. Question 6 |
|  | How satisfied were you with the pain relief from the epidural? | ERCQ. Question 7 |
|  | The labour went nearly exactly as I had hoped that it would. | WOMBLSQ. Question 11 |
| The effect of epidural is just as what I have expected. | How did the epidural compare to your expectations? | ERCQ. Question 13 |
|  | Labour was just a matter of doing what I was told by my carers. | WOMBLSQ. Question 30 |
|  | All in all, were the service you received during your stay at the delivery ward what you expect? | PreMaPEQ. Question 68 |
|  | The delivery went almost completely as I had hoped that it would. | WOMBLSQ. Question 17 |
| I was well taken care of by the staffs in the labor room and the delivery room, there is no need for improvement. | My carers couldn’t have been more helpful. | WOMBLSQ. Question 32 |
| I was treated politely and with respect by the healthcare personnel in the labor room and the delivery room. | Were you treated politely and with respect bt the health personnel at the delivery room? | PreMaPEQ. Question 52 |
|  | All my carers treated me in the most friendly and courteous manner possible. | WOMBLSQ. Question 27 |
| Overall, I am satisfied with my experience in the labor room and the delivery room. | All in all, were you satisfied with the service you received during your stay at the delivery ward? | PreMaPEQ. Question 67 |

PreMaPEQ: The Pregnancy and Maternity Care Patients’ Experiences Questionnaire[1]

P-BESS: The Preterm Birth Experience and Satisfaction Scale[2]

WOMBLSQ: The Women’s Views of Birth Labor Satisfaction Questionnaire[3, 4]

* ERCQ: The epidural related complications Questionnaire, which was proceed from a study of epidural analgesia informed consent issues[5]

1. Sjetne IS, Iversen HH, Kjollesdal JG. A questionnaire to measure women's experiences with pregnancy, birth and postnatal care: instrument development and assessment following a national survey in Norway. *BMC Pregnancy Childbirth.* 2015; 15:182.

2. Sawyer A, Rabe H, Abbott J, Gyte G, Duley L, Ayers S *et al*. Measuring parents' experiences and satisfaction with care during very preterm birth: a questionnaire development study. *BJOG.* 2014; 121(10):1294-1301.

3. Marin-Morales D, Carmona-Monge FJ, Penacoba-Puente C, Olmos Albacete R, Toro Molina S. Factor structure, validity, and reliability of the Spanish version of the Women's Views of Birth Labour Satisfaction Questionnaire. *Midwifery.* 2013; 29(12):1339-1345.

4. Smith LF. Development of a multidimensional labour satisfaction questionnaire: dimensions, validity, and internal reliability. *Qual Health Care.* 2001; 10(1):17-22.

5. Pattee C, Ballantyne M, Milne B. Epidural analgesia for labour and delivery: informed consent issues. *Can J Anaesth.* 1997; 44(9):918-923.
