# Supplementary material for: Satisfaction in parturients receiving epidural analgesia after prenatal shared decision-making intervention: a prospective, before-and-after cohort study
Source: BMC Pregnancy Childbirth. 2020 Jul 20;20:413. doi: 10.1186/s12884-020-03085-6 (PMC7370438; doi:10.1186/s12884-020-03085-6)
Supplement: Supplementary file 2 — Additional file 2. The Version 1 questionnaire. This is the pre-testing questionnaire administered to the parturients, and the English version is only translated for the publication of this research. [file 12884_2020_3085_MOESM2_ESM.docx]

|  | Strongly agree | Agree | Neither agree nor disagree | Disagree | Strongly disagree |
| --- | --- | --- | --- | --- | --- |
| **Healthcare Communication** | | | | | |
| I received sufficient information from healthcare personnel about options for pain relief before my labor course started. (CQ1) |  |  |  |  |  |
| I received sufficient information about options for pain relief during my stay at the labor room. (CQ2) |  |  |  |  |  |
| I was treated politely and with respect by the healthcare personnel in the labor room. (CQ3) |  |  |  |  |  |
| **Labor Pain** | | | | | |
| I should have been offered more to relief my labor pain. (PQ1) |  |  |  |  |  |
| I received excellent pain relief during labor. (PQ2) |  |  |  |  |  |
| More pain relief would have made my labor easier. (PQ3) |  |  |  |  |  |
| **Overall Satisfaction** | | | | | |
| I was well taken care of by the staffs in the labor room and the delivery room, there is no need for improvement. (SQ1) |  |  |  |  |  |
| I am satisfied with only one or two things regarding the labor care that I received. (SQ2) |  |  |  |  |  |
| Overall, I am satisfied with my experience in the labor room and the delivery room. (SQ3) |  |  |  |  |  |
| **Access to Information** | | | | | |
| I understand what I might have encountered during and after receiving the epidural injection. (IQ1) |  |  |  |  |  |
| I understand I may temporarily experience headaches after the injection. (IQ2) |  |  |  |  |  |
| I understand I may temporarily experience low blood pressure after receiving epidural. (IQ3) |  |  |  |  |  |
| I understand I may temporarily have trouble urinating after receiving epidural. (IQ4) |  |  |  |  |  |
| I understand I may temporarily not be able to walk due to leg numbness after receiving epidural. (IQ5) |  |  |  |  |  |
| **Decision** | | | | | |
| I feel that I received the information that I needed in order to make a decision about having an epidural. (DQ1) |  |  |  |  |  |
| Even though I was distressed during labor, I feel I was able to fully understand the information given to me by the anesthesiologist. (DQ2) |  |  |  |  |  |
| I am satisfied with the information given to me by the anesthesiologist giving me my epidural. (DQ3) |  |  |  |  |  |
| There is no risk at all in receiving epidural. (DQ4) |  |  |  |  |  |
| **Expectation and Reality** | | | | | |
| The effect of epidural is just as what I have expected. (RQ1) |  |  |  |  |  |
| I think my epidural is effective. (RQ2) |  |  |  |  |  |
| I received sufficient pain relief during delivery. (RQ3) |  |  |  |  |  |
| The labor process is just as what I have expected. (RQ4) |  |  |  |  |  |
