# Supplementary material for: Satisfaction in parturients receiving epidural analgesia after prenatal shared decision-making intervention: a prospective, before-and-after cohort study
Source: BMC Pregnancy Childbirth. 2020 Jul 20;20:413. doi: 10.1186/s12884-020-03085-6 (PMC7370438; doi:10.1186/s12884-020-03085-6)
Supplement: Supplementary file 3 — Additional file 3. Exploratory factor analysis results. Exploratory factor analysis results of all the version 1 questionnaire questions after pre-testing. [file 12884_2020_3085_MOESM3_ESM.docx]

**Additional file 3:** Exploratory factor analysis of the version 1 questionnaire

| Items* | | | | | |
| --- | --- | --- | --- | --- | --- |
|  | Factor loading calculation | | | | |
|  | 1 | 2 | 3 | 4 | 5 |
| DQ3 | .924 |  | .135 | .128 |  |
| DQ2 | .924 |  | .135 | .128 |  |
| IQ5 | .920 |  | .198 |  | .131 |
| IQ1 | .765 | .104 | .149 |  | .249 |
| DQ1 | .681 | .153 | .110 | .250 | .154 |
| RQ2 | .101 | .878 | -.104 |  |  |
| PQ2 | .125 | .848 |  |  | .124 |
| RQ3 |  | .840 | -.130 | .161 | .181 |
| RQ1 |  | .737 | .547 |  |  |
| SQ2 |  | -.615 | .202 | .523 |  |
| RQ4 |  | .614 | .475 | .164 |  |
| IQ4 | .380 |  | .836 |  |  |
| IQ3 | .397 |  | .835 |  |  |
| IQ2 | .375 |  | .832 |  |  |
| PQ3 | -.241 | -.390 | .670 |  | .136 |
| PQ1 | -.287 | -.474 | .510 | .353 |  |
| CQ2 | .514 |  | -.107 | .790 | .132 |
| CQ1 | .577 |  |  | .733 | .172 |
| DQ4 | -.115 | .381 | .241 | .545 |  |
| SQ3 | .305 | .162 |  |  | .838 |
| SQ1 | .145 |  |  | .306 | .787 |
| CQ3 | .298 |  |  | .497 | .499 |

*The abbreviation of each item comes from the version 1 questionnaire (Additional file 2) in our research
