# Supplementary material for: Satisfaction in parturients receiving epidural analgesia after prenatal shared decision-making intervention: a prospective, before-and-after cohort study
Source: BMC Pregnancy Childbirth. 2020 Jul 20;20:413. doi: 10.1186/s12884-020-03085-6 (PMC7370438; doi:10.1186/s12884-020-03085-6)
Supplement: Supplementary file 4 — Additional file 4. The Version 2 questionnaire. This is the English version of Version 2 questionnaire, whose categories were based on the above-methodology chapter-mentioned statistical method. [file 12884_2020_3085_MOESM4_ESM.docx]

**Additional file 4:** Version 2 questionnaire.

| Categories based on the above-methodology chapter-mentioned statistical method | Factor loadings |
| --- | --- |
| Category 1 |  |
| Even though I was distressed during labor, I feel I was able to fully understand the information given to me by the anesthesiologist. | 0.924 |
| I am satisfied with the information given to me by the anesthesiologist giving me my epidural. | 0.924 |
| I understand I may temporarily not be able to walk due to leg numbness after receiving epidural. | 0.920 |
| I understand what I might have encountered during and after receiving the epidural injection. | 0.765 |
| I feel that I received the information that I needed in order to make a decision about having an epidural. | 0.681 |
| Category 2 |  |
| I think my epidural is effective. | 0.878 |
| I received excellent pain relief during labor. | 0.848 |
| I received sufficient pain relief during delivery. | 0.840 |
| Pain relief is almost the same as I expected. | 0.737 |
| The labor process is as I expected. | 0.614 |
| Category 3 |  |
| I understand I may temporarily experience headaches after the injection. | 0.836 |
| I understand I may temporarily experience low blood pressure after receiving epidural. | 0.835 |
| I understand I may temporarily have trouble urinating after receiving epidural. | 0.832 |
| I should have been offered something more to relief my labor pain. | 0.670 |
| More pain relief would have made my labor easier. | 0.510 |
| Category 4 |  |
| I received sufficient information about options for pain relief, both epidural and non-pharmacological managements, during my stay at the labor room. | 0.790 |
| I received sufficient information from healthcare personnel about options for pain relief, both epidural and non-pharmacological managements, before my labor course started. | 0.733 |
| There is no risk at all to reduce labor pain. | 0.545 |
| I am satisfied with just one or two things about the labor care that I received. | 0.523 |
| Category 5 |  |
| Overall, I am satisfied with my experience in the labor room and the delivery room. | 0.838 |
| I was well taken care of by the staffs in the labor room and the delivery room, there is no need for improvement. | 0.787 |
| I was treated politely and with respect by the healthcare personnel in the labor room and the delivery room. | 0.499 |
