# Supplementary material for: Satisfaction in parturients receiving epidural analgesia after prenatal shared decision-making intervention: a prospective, before-and-after cohort study
Source: BMC Pregnancy Childbirth. 2020 Jul 20;20:413. doi: 10.1186/s12884-020-03085-6 (PMC7370438; doi:10.1186/s12884-020-03085-6)
Supplement: Supplementary file 5 — Additional file 5. Exploratory factor analysis results. Exploratory factor analysis results of all the version 3 questionnaire questions after pre-testing. [file 12884_2020_3085_MOESM5_ESM.docx]

**Additional file 5:** Exploratory factor analysis results of all the version 3 questionnaire items after pre-testing.

|  | | | | | |
| --- | --- | --- | --- | --- | --- |
|  | Factor loading calculation | | | | |
|  | 1 | 2 | 3 | 4 | 5 |
| IQ1 | .771 | .153 | .041 | .090 | .279 |
| DQ1 | .663 | .152 | .203 | .333 | .083 |
| DQ3 | .901 | .216 | .011 | .226 | .077 |
| PQ2 | .190 | .022 | .881 | -.009 | .092 |
| RQ1 | -.048 | .583 | .703 | -.127 | .115 |
| RQ2 | .121 | -.052 | .902 | .007 | .027 |
| RQ3 | -.080 | -.061 | .880 | .207 | .114 |
| IQ2 | .286 | .892 | -.046 | -.035 | .042 |
| IQ3 | .255 | .932 | .009 | .127 | -.004 |
| IQ4 | .241 | .929 | .024 | .069 | .013 |
| IQ5 | .900 | .264 | .078 | .175 | .105 |
| CQ1 | .460 | .059 | .021 | .801 | .122 |
| CQ2 | .390 | -.012 | .014 | .879 | .040 |
| DQ2 | .901 | .216 | .011 | .226 | .077 |
| SQ1 | .119 | -.065 | .113 | .343 | .779 |
| CQ3 | .091 | .082 | .087 | .790 | .363 |
| SQ3 | .274 | .109 | .142 | .075 | .865 |

The above items were based on the version 1 questionnaire (Additional file 2) in our research
